# How to identify depression and anxiety in children and adolescents

**DOI:** 10.1016/j.jped.2025.101462

**Published:** 2025-11-09

**Authors:** Coraima A. Linan, Gibsi MP Rocha, Marta K. Lucion

**Affiliations:** Pontifícia Universidade Católica do Rio Grande do Sul, Faculdade de Medicina, Departamento de Neurociências, Porto Alegre, RS, Brazil

**Keywords:** Adolescent, Anxiety, Child, Depression, Diagnosis

## Abstract

**Objective:**

To review the epidemiology, diagnostic criteria, and therapeutic options for the most prevalent anxiety and depressive disorders in childhood and adolescence.

**Sources of data:**

A non-systematic review was conducted in the PubMed, SciELO, and Google Scholar databases. Diagnostic criteria were defined according to the Diagnostic and Statistical Manual of Mental Disorders (DSM-5-TR) and updated textbooks on child and adolescent psychiatry.

**Summary of the data:**

Diagnosis requires a comprehensive clinical assessment, considering age-specific manifestations and differentiating expected developmental emotional responses from pathological conditions.

**Conclusions:**

Management relies on psychoeducation and psychotherapeutic interventions—particularly cognitive-behavioral therapy—and, in moderate to severe cases, pharmacotherapy with selective serotonin reuptake inhibitors, preferably combined with psychotherapy. Early identification, especially at the pediatric care level, is essential to reduce morbidity, prevent recurrence, and promote healthy development.

## Introduction

Anxiety disorders (AD) and depressive disorders (DD) represent the most prevalent mental health problems in childhood and adolescence. It is estimated that 10 to 20 % of this population will experience some form of anxiety or depression over the course of their lifetime [1, 2, 3]. Furthermore, anxiety and depression are among the top three mental illnesses in the global burden of disease assessment across all age groups. In the group of people aged 15 and older, they rank second and third, respectively, among all diseases analyzed by the Global Burden of Disease study [2]. Major depressive disorder (MDD), in particular, is the third leading cause of disability-adjusted life years, demonstrating its significant impact on the global burden of disease in this age group [2, 3, 4, 5, 6].

The World Health Organization (WHO) estimates that depression is one of the leading causes of illness and disability in children and adolescents, indicating an increase of approximately 60 % in cases over the last decade [7, 8, 9]. AD and DD significantly impact an individual's overall functioning, affecting school performance, peer socialization, family relationships, emotional development, and future employment [3,10]. Children and adolescents with internalizing symptoms are at increased risk of school dropout, social isolation, early substance use, and family conflict [3,6,11].

Early identification and appropriate management of these conditions are essential to reduce their negative long-term impacts, promoting the resumption of healthy development and improving prognosis. In this context, understanding the clinical manifestations, diagnostic strategies, and possible treatments is a fundamental skill for healthcare professionals, especially those working on the front lines of pediatric care.

Therefore, this article aims to review the epidemiology, diagnostic criteria, and therapeutic options for the main AD and DD in childhood and adolescence.

## Methods

A non-systematic review was conducted in the PubMed, SciELO, and Google Scholar databases, using the terms: “child anxiety,” “depression,” “youth,” “adolescence,” “anxiety disorder,” “anxiety and childhood,” and “anxiety disorders.” To define the diagnostic criteria, established references were consulted, such as the Diagnostic and Statistical Manual of Mental Disorders (DSM-5-TR) and updated textbooks on child and adolescent psychiatry.

### Epidemiology

AD and DD are the most common mental health problems in the child and adolescent population [2,12]. Anxiety is estimated to be the most prevalent mental disorder in this age group, with symptomatic prodromes observed as early as age 6 [13], affecting approximately 117 million children and adolescents. Among the most prevalent AD in childhood and adolescence are separation anxiety disorder (7.6 %), social anxiety disorder (9.1 %), generalized anxiety disorder (3 %), and panic disorder with agoraphobia (2.3 %) in the general population [3,6,13].

The incidence of MDD among prepubescent children ranges from 1 to 3 %. In adolescence (12–18 years), this incidence increases significantly to approximately 4 to 8 % per year, being more common in girls after adolescence [4].

Genetic and environmental factors are involved in the risk of depression and anxiety [9,14, 15, 16]. Having a caregiver with a psychiatric disorder represents both a genetic and environmental risk, highlighting the importance of parental treatment for healthy child development [3,14,16, 17, 18, 19]. Environmental risk factors include withdrawn temperament, social vulnerability, family dysfunction, early trauma, academic difficulties, being a victim of bullying and/or violence, sleep disorders, excessive screen use, substance abuse, functional and structural changes in brain circuits, and the presence of chronic disease [3,4,9,13, 14, 15, 16, 17, 18, 19, 20, 21].

Several protective factors have been shown to be effective in preventing and mitigating anxiety and depression symptoms, especially in children and adolescents. Adequate family functioning is one of the main protective factors, as it provides an environment characterized by emotional support, stability, and effective communication. Furthermore, regular physical exercise plays a significant role in promoting mental health, being associated with reduced stress, improved mood, and increased feelings of well-being [14,16].

### Diagnosis

The diagnosis of AD and DD is made through a clinical evaluation, which requires the integration of information from multiple sources, including patients, parents, and, when possible, the school or other people close to the young person. Internalizing disorders are characterized by intrapsychic manifestations, which are not always verbalized to caregivers. Therefore, close observation of changes in a child's or adolescent's functioning can help identify psychological distress — for example, when a child loses interest in playing or begins to repeatedly visit the school nurse due to nonspecific complaints [12].

#### Diagnosis of anxiety

In normal development, there are expected periods of heightened anxiety, which serve the evolutionary purpose of preparing the child to face future challenges. However, these periods are not usually excessive in relation to stimulation, nor do they persist beyond what is expected (>6 months). In infants (0–18 months), anxiety around strangers, caused by separation from attachment figures and loud noises, is common. In preschool children, fears related to the dark, nightmares, monsters, unknown people, and ghosts predominate. From the age of 4, fears of physical injury, illness, or the possibility of close family members being affected may emerge. In preadolescence and adolescence, the most frequent fears are associated with social situations, physical appearance, performance, and competence in different domains [3,22,23].

The central characteristic of AD is the presence of fear and perception of threat, which induce significant clinical distress. Depending on the anxiety subtype, this response is directed toward a specific stimulus, which is generally harmless or of small magnitude compared to the actual reaction, but which triggers persistent avoidance or anxious anticipation of suffering. In children, fear and threat often manifest through multiple nonspecific physical symptoms, such as headache, diffuse abdominal pain, nausea, vomiting, diarrhea, subjective feelings of tension, muscle stiffness, and insomnia [12,22,24].

Anxiety symptoms manifest in a dimensional manner, ranging from an expected reaction to stress to exacerbated reactions that constitute a disorder. An anxiety disorder is considered when symptoms persist for at least 6 months, are highly intense, and cause harm to the individual. Table 1 describes the main symptoms of the most prevalent ADs in childhood and adolescence [12,22,24].

**Table 1:** Characteristics of anxiety disorders in the child and adolescent population.

Disorder	Main characteristics
Separation anxiety disorder	Excessive and inappropriate fear or anxiety about separation from attachment figures; persistent worry about harm to or loss of attachment figures; reluctance to leave the home or sleep alone; physical symptoms of distress when separation occurs.
Social anxiety disorder	Intense and persistent fear of social situations involving possible evaluation by others; concern about humiliation or rejection; avoidance of social interactions; impairment in academic and social functioning.
Generalized anxiety disorder	Excessive anxiety and worry, difficult to control, in relation to multiple areas of life; associated symptoms such as restlessness, fatigue, difficulty concentrating, irritability, muscle tension, and sleep disturbances.
Panic disorder	Panic attacks are characterized by intense autonomic symptoms such as palpitations, dyspnea, dizziness, and fear of losing control. Symptoms are recurrent and unexpected, accompanied by persistent worry about new episodes or their consequences, and behavioral avoidance.
Agoraphobia	Intense fear or anxiety in two or more situations, such as using public transportation, being in open spaces, being in closed spaces, standing in lines or crowds, and leaving home alone; avoidance of these situations due to concerns about difficulty escaping or unavailability of help; significant impact on functioning.

***Warning signs to suspect anxiety:*** [12]

• Inhibited behavior
• Withdrawal and reluctance toward strangers
• Difficulty fitting in with strangers or peers
• Reduced smiling and speech
• Seeking closeness with a caregiver
• Limited eye contact or "shy look"

#### Differential diagnosis of AD

Other disorders can also present with manifestations of anxiety and excessive worry, such as obsessive-compulsive disorder (OCD), post-traumatic stress disorder (PTSD), attention deficit hyperactivity disorder (ADHD), schizophrenia, and other psychotic disorders [22,24].

Clinical conditions that can present with anxiety-like symptoms include hypothyroidism or hyperthyroidism, cardiac arrhythmias (such as paroxysmal supraventricular tachycardia), epilepsy, hypoglycemia, and vestibular disorders [22].

#### Diagnosis of depression

Like anxiety, depressive symptoms are part of normal development. Depressive reactions to certain events and frustrations are important in the development of the psyche. The difference between depressive reactions and a depressive episode is related to intensity (depression is considered when symptoms are present most of the day, on most days), duration, and level of impairment. Table 2, Table 3 describe the diagnostic criteria for MDD [8,24] and its manifestations in each age group [8].

**Table 2:** Symptoms and diagnostic criteria for depression.

Category	Criterion	Observation
General conditions	- Presence of ≥ **5 symptoms** in the same 2-week period, representing a change from previous functioning. - At least **one of the symptoms must be depressed mood or anhedonia**. – The symptoms must not be explained by another medical condition.	Irritability is considered nonspecific, as it can be present in ASD, ODD, OCD, BAD, ADHD.
Nuclear symptoms (required ≥ 1)	1. Subjective feeling of sadness, unhappiness, and/or irritability. 2. Anhedonia/loss of pleasure in previously pleasurable activities.	One of the two must be present in the diagnosis.
Additional symptoms (required ≥ 4)	1. Significant change in appetite or weight (without dieting). 2. Insomnia or hypersomnia. 3. Psychomotor agitation or retardation. 4. Fatigue or loss of energy. 5. Feelings of worthlessness or inappropriate guilt. 6. Reduced concentration, impaired reasoning, frequent indecisiveness, recurrent thoughts of death or suicide.	At least four of these symptoms must be present in the current episode.

BAD, bipolar affective disorder; ADHD, attention deficit hyperactivity disorder; ASD, autism spectrum disorder; OCD, obsessive-compulsive disorder; ODD, oppositional defiant disorder.

**Table 3:** Differences in the clinical presentation of depression by age group.

Age group	Most frequent clinical features
Prepubertal	- Irritability (tantrums, disobedience) - Reactive affect* - Frequent comorbidities (anxiety, ADHD, behavioral problems) - Somatic complaints
Adolescents	- Irritability (resentments, hostility, outbursts of anger) - Reactive affect* - Hypersomnia or insomnia - Changes in appetite and weight - Somatic complaints - Extreme sensitivity to rejection (distorted interpretations of criticism or contempt), making relationships difficult
Children	- Anhedonia - Absence of affective reactivity - Psychomotor agitation or retardation - Diurnal mood variation (worse in the morning)

ADHD, attention deficit hyperactivity disorder.

There are some differences in the manifestation of a depressive episode in children and adolescents compared to adults. Children and adolescents may present with both depressed mood, with sadness and discouragement, and irritable mood. Reactive affect is also common: at times, adolescents may show interest and willingness in pleasurable activities, but outside of these situations, they remain symptomatic most of the time. In addition, autonomic and/or somatic symptoms may occur, such as lack of energy, loss or increase in appetite, sleep disturbances (especially insomnia), and widespread pain [8,9,25].

The most commonly reported symptoms by adolescents during a depressive episode are sadness, social isolation, and loneliness [26]. In patients with mood disorders, thoughts of death and self-harm should always be investigated (Table 2, Table 3).

***Warning signs of depression:***

• Chronic boredom or loss of interest in previously enjoyable activities (e.g., dropping out of sports, dance, or music lessons)
• Social withdrawal, or no longer wanting to hang out with friends
• Resistance to going to school
• Decreased academic performance
• Complaints of frequent physical symptoms without an organic cause, such as "feeling unwell," headaches, or epigastric pain
• Behavioral problems (defiant behavior, running away from home, bullying peers)
• Use of psychoactive substances

#### Differential diagnosis of DD

Some disorders can also present with depressive symptoms, such as persistent depressive disorder (dysthymia in the DSM-IV), adjustment disorder, disruptive mood dysregulation, and bipolar disorder [8,9,24].

### Comorbidities

Approximately 10 to 15 % of adolescents diagnosed with anxiety or depression have other comorbid psychiatric disorders. Untreated AD increases the risk of depressive episodes by up to four times and tends to have a chronic course. It is estimated that, without adequate intervention, up to 70 % of cases experience recurrence of symptoms within 5 years.

These conditions are associated with a wide range of complications, including other depressive and anxiety disorders, PTSD, OCD, conduct disorder, ADHD, substance abuse, learning disabilities, eating disorders, and suicidal and non-suicidal self-harm behaviors. The latter constitute, respectively, the second and third leading causes of mortality among adolescents [3,8,9,15,25,27,28].

### Treatment

The choice of treatment is directly related to the severity of the condition. In mild and moderate cases of both AD and DD, the first indication is psychoeducation combined with psychotherapy. Cognitive-behavioral therapy (CBT) is the most studied modality and has robust evidence of efficacy in the treatment of anxiety and depression in childhood and adolescence. When there is a history or risk of suicide and/or self-harm, psychotic symptoms, and panic attacks with agoraphobia, pharmacotherapy is often indicated [3,8,9,12,13,22,23,27,29,30].

The combination of medication and CBT is recommended for patients with more severe conditions. Combined treatment has been shown to be more beneficial than therapy or medication alone, with reduction and control of anxiety and depressive symptoms in almost 70 % of patients within 8 weeks. Selective serotonin reuptake inhibitors (SSRIs) are the first-line treatment of choice for both depression and anxiety [3,8,9,12,13,22,27,29,30].

Table 4 shows the medications approved by the Food and Drug Administration for the treatment of these disorders [8,9,29,30]. Fluoxetine is the SSRI with the most studies and the highest level of evidence regarding efficacy and safety, followed by sertraline and escitalopram. Other studied second- and third-line options include fluvoxamine, duloxetine, venlafaxine, and paroxetine. Prepubertal children tend to have a greater placebo effect compared to adolescents, in addition to a lower pharmacological response. Tricyclic antidepressants are not routinely recommended due to their multiple adverse effects and the need for rigorous electrocardiographic and laboratory monitoring [3,8,9,12,13,22,27,29,30].

**Table 4:** Antidepressants in children and adolescents.

Drug	Indication	Age	Initial dose	Recommended dose
Fluoxetine	Depression, OCD	> 8 (depression) > 7 (OCD)	10 mg (drops: 20 mg/mL)	20–60 mg (minors: less)
Sertraline	OCD	> 6	25 mg	25–200 mg
Fluvoxamine	OCD	> 8	25 mg (increases in 4–7 days)	50–300 mg
Escitalopram	Depression, GAD	> 12 (dep.) > 7 (GAD)	5–10 mg (drops: 20 mg/mL)	5–20 mg
Paroxetine	Off label*	—	10 mg / 12.5 mg	—
Venlafaxine	Off label*	—	37.5 mg	75–150 mg (up to 300 mg in adolescents)
Duloxetine	GAD	> 7	30 mg	30–60 mg
Bupropion	Off label*, 3rd/4th line ADHD	—	150 mg	150–300 mg
Vortioxetine	Off label*	Adolescent	10 mg	10–20 mg
Amitriptyline	Depression	> 12	10 mg TID	50–100 mg

GAD, generalized anxiety disorder; ADHD, attention deficit hyperactivity disorder; TID, 3 times a day; OCD, obsessive-compulsive disorder.

* Use not specified in the package insert.

It is important to highlight the possibility of increased suicidality during the first weeks of antidepressant treatment. Although they can intensify thoughts of death, there is no evidence of an increase in completed suicides. The benefits of treatment outweigh the risks, as untreated depression significantly increases the risk of suicide. Close monitoring is recommended, with systematic clinical reviews during medication introduction and at each dose adjustment [8,9,29,30].

The success of pharmacological treatment depends on adequate adherence, that is, daily use and maintenance for at least 6 months after remission of initial symptoms. Medication withdrawal should be done gradually [3,8,9,12,13,22,27,29].

## Role of the pediatrician

Pediatricians play a fundamental role in the initial diagnosis of depression and anxiety in childhood and adolescence, as they are often the healthcare professional with the most regular contact with this population — whether through routine checkups, vaccinations, or growth and development monitoring. This close relationship allows pediatricians to understand the child's history, family dynamics, and school performance, facilitating early identification of subtle changes in behavior, mood, or social functioning patterns, which often represent the first signs of these disorders.

Early detection is essential to prevent symptoms from becoming chronic and enable more effective interventions, reducing the negative impact of these conditions on the emotional, academic, and social development of young people. It is the pediatrician's responsibility to make timely referrals to mental health professionals when necessary [3,22]. Therefore, it is imperative that they be properly trained to accurately and early recognize the clinical manifestations of AD and DD in children and adolescents — a key measure to reduce the risk of significant functional impairment and the chronicity of the condition if not treated appropriately [3,22].

**Pearls for Home**

• Main criteria for therapeutic choice: effectiveness and safety (minimum effective dose).
• SSRIs are the safest class of drugs.
• Fluoxetine has the best evidence of effectiveness in children and adolescents; start with 10 mg/day.
• If well tolerated, increase to 20 mg/day after one week.
• A dose of 20 mg/day is usually sufficient in prepubertal children.
• Adolescents may require 30–40 mg/day, if well tolerated.
• In case of intolerance or contraindication to fluoxetine, consider other SSRIs (sertraline or escitalopram).

## Conclusion

Due to the multimodal factors involved in internalizing disorders, the presence of one of these conditions increases the likelihood of the other developing by up to four times during childhood and adolescent development. Currently, the most effective strategy is undoubtedly prevention, through early identification of children and adolescents at socio-environmental or hereditary risk, enabling the provision of appropriate treatment based on the clinical picture.

## Statement on the use of generative AI and AI-assisted technologies in the writing process

Disclosure: During the preparation of this manuscript, the authors used ChatGPT for spell checking. After using this tool/service, the authors reviewed and edited the content as needed, assuming full responsibility for the content of the publication.

## Sources of funding

None.

## Authors’ contributions

Coraima Anaya Linan: Data processing, formal analysis, investigation, methodology, project administration, validation, visualization, writing – original draft, and writing – review & editing.

Gibsi Maria Possapp Rocha: Conception, data processing, formal analysis, investigation, methodology, project administration, supervision, validation, visualization, writing – original draft, and writing – review & editing.

Marta Knijnik Lucion: Conception, data processing, formal analysis, investigation, methodology, project administration, supervision, validation, visualization, writing – original draft, and writing – review & editing.

**Data availability statement:** The data that support the findings of this study are available from the corresponding author.

## Conflicts of interest

The authors declare no conflicts of interest.
